# Effects of Annealing Processes on Microstructure and Properties of FeNi-Based Amorphous Alloy

**DOI:** 10.3390/ma18133172

**Published:** 2025-07-04

**Authors:** Chenglong Sun, Mengen Shi, Xinyu Wang, Daying Deng, Weihuo Li

**Affiliations:** School of Materials Science and Engineering, Anhui University of Technology, Maanshan 243032, China; clongsun@163.com (C.S.); genm612@163.com (M.S.); 13053084071@163.com (X.W.); 15695556918@163.com (D.D.)

**Keywords:** transverse magnetic annealing, longitudinal magnetic annealing, FeNi-based amorphous nanocrystals, soft magnetic properties, mechanical properties

## Abstract

The present experiment is aimed at investigating the changes in the properties of an FeNiCBCo amorphous alloy after different stress relief annealing. It was established that, under equivalent temperature and time conditions, the strip that underwent no magnetic field annealing exhibited the maximum *B*_s_ of 1.09 T. The soft magnetic properties were found to be marginally enhanced by the transverse magnetic treatment, and the coercivity was notably reduced from 10.15 to 0.27 A/m after the longitudinal magnetic treatment. Furthermore, it was determined that, subsequent to the longitudinal magnetic treatment and the annealing treatment with no magnetic field, the strip exhibited enhanced mechanical properties due to the precipitation of the second phase A1 FeNi nanoparticles within the strip. In contrast, the transverse magnetic treatment significantly improved the strength of the alloy. Additionally, the strip demonstrated superior mechanical properties, while the strength of the alloys with the transverse magnetic treatment was significantly increased. This study demonstrates that transverse magnetic treatment can evidently enhance the strength, and magnetic field-free and longitudinal magnetic annealing treatments improve the soft magnetic properties, of amorphous alloys while maintaining good mechanical properties.

## 1. Introduction

In recent years, the development of third-generation wide-band semiconductors has led to significant advancements in the power capabilities of power electronic devices, thereby placing greater emphasis on the performance of inductive components. This has resulted in an increased demand for soft magnetic alloys, which play a pivotal role in power conversion, magnetic induction and magnetic storage. A series of new amorphous alloy systems, including Fe-based [1], Ni-based [2], Co-based [3], Mg-based [4], and FeNi-based [5], amongst others, have been developed in succession, exhibiting enhanced amorphous formation capabilities and soft magnetic properties. Among these, the FeNi-based amorphous alloy system has garnered particular attention. Nanocrystalline soft magnetic alloys have attracted significant attention due to their advantageous properties, including their low coercivity (*H*_c_), high permeability (*μ*_e_), and low core loss (*P*_c_) [6,7,8,9]. These alloys find wide application in transformers, magnetic heads, magnetic sensors, and magnetic shields, among other applications [10,11,12,13,14], and are considered to be pivotal to enabling miniaturization, green efficiency, and low energy loss in electronic components [15,16]. However, the practical application of FeNi-based amorphous alloys is still restricted by two major issues: (1) Their low amorphous formation ability; (2) The annealing process-induced formation of hard magnetic phases, deteriorating their soft magnetic properties.

It has been reported that the partial replacement of B in Fe_48_Ni_32_B_20_ by C element results in a composition of a Fe_48_Ni_32_C_8_B_12_ alloy, which can improve the alloy’s thermal stability and glass-forming ability [17,18] and compete with element B to inhibit the precipitation of hard magnetic phases by enhancing multi-component atomic size mismatch and structural disorder [19]. According to the saturation magnetic induction, Co (1.8 T) is smaller than Fe (2.15 T), but much larger than Ni (0.61 T). The partial replacement of Ni by Co results in a new composition of a Fe_48_Ni_31_Co_1_C_8_B_12_ alloy, further improving the alloy’s magnetic properties for this study. In addition to the composition design, magnetic field heat treatment can effectively regulate the magnitude of Ku [20]. In the present work, magnetic field heat treatment was used to maximum saturation magnetization induction and minimum coercivity to develop a new soft magnetic material.

## 2. Experimental

### 2.1. Sample Preparation

Alloy ingots of the nominal composition Fe_48_Ni_31_Co_1_C_8_B_12_ were prepared by melting pure Fe (99.99 wt.%), Ni (99.99 wt.%), Co (99.99 wt.%), B (99.99 wt.%), and FeC alloys (4 wt.% C) in a high-purity argon atmosphere. In order to ensure the homogeneity of the composition, the melting was repeated four times for each alloy ingot. Each ingot was remelted under a vacuum to prepare an amorphous strip using a single Cu roller with a line speed of 36.7 m/s—that is to say, using the melt-spinning technique. The melt-spun strip displayed external dimensions of a width of about 1 mm and a thickness of 25 μm.

Different annealing processes were carried out on the melt-spun samples. These are conventional annealing (CA), transverse magnetic annealing (TA), and longitudinal magnetic annealing (LA), which were used in a high vacuum (3 × 10^−3^ Pa) isothermal tubular furnace at 420 °C for 5 min. To be specific, an applied magnetic field of 0.5 T was oriented either parallel to the longitudinal or perpendicular to the transverse of the strip.

### 2.2. Sample Characterization

Microstructural analysis was carried out on the melt-spun and annealed samples. X-ray diffractometry (XRD, D8-ADVANCE, Tokyo, Japan) with Cu Kα radiation (λ = 1.5418 Å) was used for phase analysis. A transmission electron microscope (TEM, JEM-F200, Tokyo, Japan) equipped with EDS was used for structure and composition information. The microstructural data were acquired in STEM mode with the following parameters: probe current: 50 pA (semi-convergence angle 25 mrad), probe size: 0.2 nm (aberration-corrected probe), and step size: 0.5 nm (dwell time 10 μs/pixel).

The coercivity and saturation magnetization induction of the melt-spun and annealed samples were tested using a Direct AC Magnetization Characterization Analyzer (B-H, CURNE TRACER, Tokyo, Japan) and a Vibrating Sample Magnetometer (VSM, Micro Sense E27, Lowell, MA, USA). The hardness and modulus of the melted and annealed samples were tested using a 3 × 4 dot-matrix dotting machine (KLA Corporation, Nano Indenter G200, Milpitas, CA, USA) with a standard Berkovich diamond material indenter. A scanning Electron Microscope (SEM, TESCAN MIRA 3, Brno, Czech Republic) was used to observe nanoindentation and fracture morphology.

## 3. Result and Discussion

**Figure 1:**
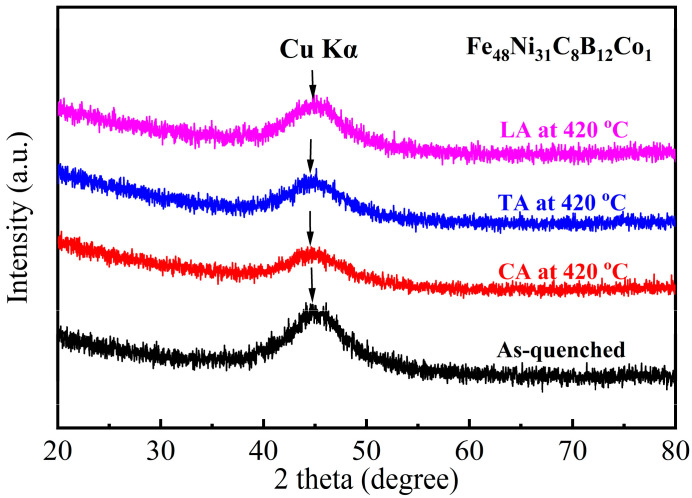
XRD patterns of as-quenched Fe_48_Ni_31_Co_1_C_8_B_12_ alloy samples subjected to conventional annealing and magnetic field annealing.

**Figure 2:**
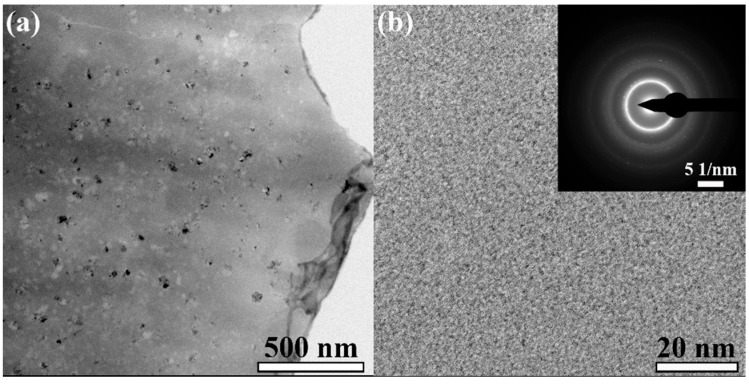
TEM images of CA sample: (**a**) Low magnification morphology; (**b**) High magnification morphology with an insert of diffraction rings.

The XRD patterns obtained from the as-quenched and annealed samples are shown in Figure 1. Obviously, there is an amorphous halo characteristic for the melt-spun sample. In comparison, the samples annealed at 420 °C also display a similar hump peak as the as-quenched sample. It is hard to identify whether nanocrystallization occurs in the annealed samples. TEM analysis was carried out to obtain more structural information. As shown in the TEM images of Figure 2, the CA sample displays a certain number of nanoparticles embedded into an amorphous matrix (Figure 2a). The degree of nanocrystallization is not very high, which is supported by the amorphous ring (Figure 2b) and unclear nanocrystalline rings.

**Figure 3:**
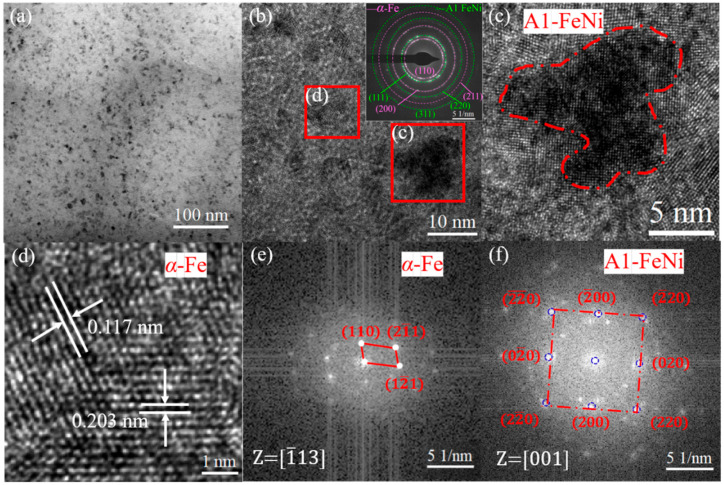
High-resolution STEM analysis of LA sample. (**a**) High magnification morphology; (**b**) Nanocrystalline diffraction rings; (**c**) Lattice fringe of A1 FeNi; (**d**) Lattice fringe of α-Fe; (**e**) FFT of α-Fe image; (**f**) FFT of A1 FeNi image.

The TEM images of Figure 3a,b show that the strips have an obvious amorphous nanocrystalline structure after longitudinal magnetic annealing treatment, and more clusters and grains precipitated in the matrix, as can be clearly seen in the low magnification morphology. The diffraction rings in the illustration were analyzed and compared, corresponding, respectively, to the (110), (200), and (211) crystal planes of the α-Fe grains and the (111), (220), and (311) crystal planes of the A1 FeNi grains. Figure 3c is an enlarged image of the same area, clearly revealing the coherent interface formed between the A1 FeNi nanocrystals and the α-Fe grains on the matrix. This is primarily due to the fact that during magnetic field annealing, the nanocrystal precursor α-Fe grains precipitate first in the matrix. Under the influence of thermal activation energy and the magnetic field, Ni atoms accumulate on the precursor α-Fe grains and gradually dissolve into Fe to form FeNi grains. The clusters and nanocrystals in the selected area were enlarged by inverse Fourier transform to calculate the crystal plane spacing and the corresponding indexed fast Fourier transform images, as shown in Figure 3d–f. The interplanar spacings are 0.203 nm and 0.117 nm, corresponding to the (110) and (211) crystal planes of α-Fe grains with a body-centered cubic structure. Through the corresponding Fourier transform, it was determined that this nanocrystal is consistent with the (220) crystal plane of the face-centered cubic structured A1 FeNi grains.

**Figure 4:**
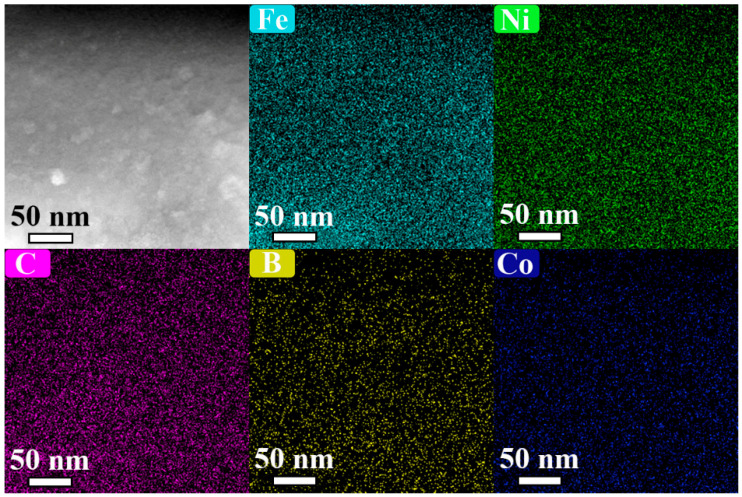
LA sample HAADF-STEM images showing morphology and elemental distribution.

It can be clearly observed in Figure 4 that the strip surface exhibits an uneven surface morphology, which is attributed to the contrast between the precipitated A1 FeNi nanocrystals (bright white particles) and the amorphous matrix during the annealing process. The elemental distribution is visualized through color mapping (Fe: cyan, Ni: green, C: purple, B: yellow, Co: blue). Overall, while thermodynamic considerations predict the elemental partitioning of Ni/Co between the FCC and BCC phases, the uniform distribution of Fe and Ni indicates that no significant elemental segregation occurred during annealing. This phenomenon is likely attributable to probe-broadening effects induced by specimen thickness variations, particularly in thicker regions where beam–sample interactions span multiple crystallites. However, localized regions exhibiting Fe and Ni enrichment are attributed to A1 FeNi nanocrystal precipitation; while the B element demonstrates a uniform distribution within the amorphous matrix, the C element exhibits negligible segregation, thereby corroborating the compositional stability of the designed alloy system.

**Figure 5:**
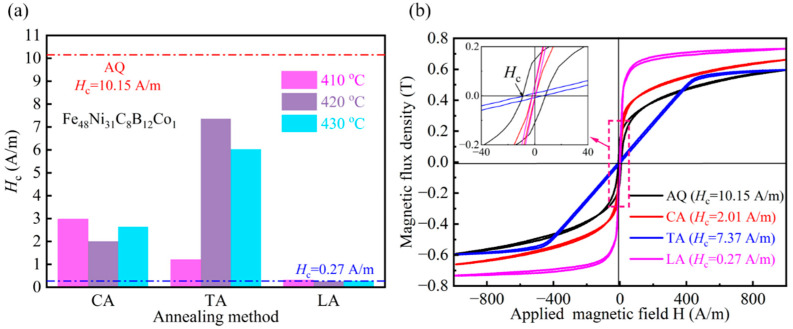
(**a**) Comparison chart of coercivity of annealed samples at different temperatures; (**b**) Hysteresis loops of annealed samples.

As can be seen in Figure 5a, the coercivity of all the annealed strips exhibited varying decreases. Although the coercivity after transverse magnetic annealing was lower than that of the quenched state, it remained significantly higher than the 2 A/m value observed in the non-magnetic field annealed sample. Notably, the coercivity (*H*_c_) decreased dramatically from 10.15 A/m to 0.27 A/m following longitudinal magnetic field annealing. This reduction is attributed to the combined effects of magnetic field orientation and thermal activation promoting magnetic moment alignment. As shown in Figure 3, STEM analysis revealed the precipitation of A1 FeNi nanocrystals during annealing, where the low magnetic crystalline anisotropy facilitated the magnetic domain wall motion [21]. Furthermore, longitudinal magnetic field-induced atomic ordering effectively reduced residual stress, further diminishing the pinning effect on the magnetic domain walls.

As shown in Figure 5b, the samples treated with CA and LA exhibited higher squareness of hysteresis loops and steeper rates of increase in magnetic flux density (dB/dH), while the hysteresis loop of TA-treated samples tended to flatten. This difference is directly related to the microstructural evolution induced by different annealing processes: CA and LA processes promote the uniform precipitation of A1 FeNi nanocrystals. The low magnetic crystalline anisotropy of the FCC structure reduces magnetostatic ‘pinning effects’, allowing magnetic moments to align easily along the external field direction, thereby enhancing permeability and loop squareness. In contrast, the TA process induces the localized enrichment of atomic clusters with higher magnetic crystalline anisotropy, which hinders the movement of domain walls. This results in a magnetization process dominated by magnetization rotation rather than wall motion, significantly reducing the rate of magnetic flux density increase and causing hysteresis loop flattening.

**Figure 6:**
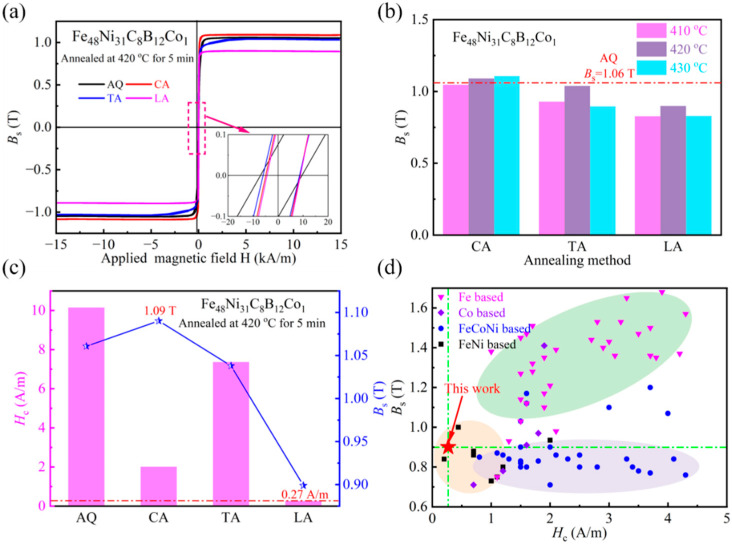
(**a**) M-H curves of different samples; (**b**) Comparative diagram of saturation magnetic induction (*B*_s_); (**c**) Comparative diagram of coercivity (*H*_c_) and *B*_s_ for different samples; (**d**) Comparison of *H*_c_ and *B*_s_ from this study with other research findings [23].

Analyzing in Figure 6a the magnetic induction intensity comparison graph and in Figure 6b the M-H curve, it can be seen that the saturation magnetic induction intensity of the two groups of magnetic field annealed strips showed a certain decrease, and that the decrease in the longitudinal magnetic treatment was more obvious. This is due to the dual action of the thermal activation energy and the magnetic field, which promotes the amorphous matrix of α-Fe and the combination of Ni atoms to form the A1 FeNi grains. A review of the literature reveals that amorphous alloys with high Fe content possess high saturation magnetic induction strength (see reference [22]). However, following magnetic field annealing, the volume fraction of α-Fe in the matrix diminishes, concomitant with the induction of magnetic anisotropy within the matrix due to the effect of the applied magnetic field. This, in turn, results in a reduction in the saturation magnetic induction strength of the alloy. As demonstrated in Figure 6c,d, the saturation magnetic induction strength of the present experimental alloy is lower than that of Fe-based amorphous alloys due to the low Fe content, but at the same time, it has a smaller coercivity. Within the same system of alloys, the untreated alloy exhibits a higher *B*_s_ (1.09 T); although there is a slight decrease, it remains in the range of 0.9 T or more, whereas other alloys exhibit a more moderate level. Concurrently, after treatment, the alloy demonstrates a lower *H*_c_ (0.27 A/m) than the other components. In summary, this experiment focused on the preparation and treatment of strips, which exhibited satisfactory comprehensive soft magnetic properties. Subsequent composition adjustments were made to enhance the saturation magnetic induction strength of the system, thereby expanding its application scenarios.

**Figure 7:**
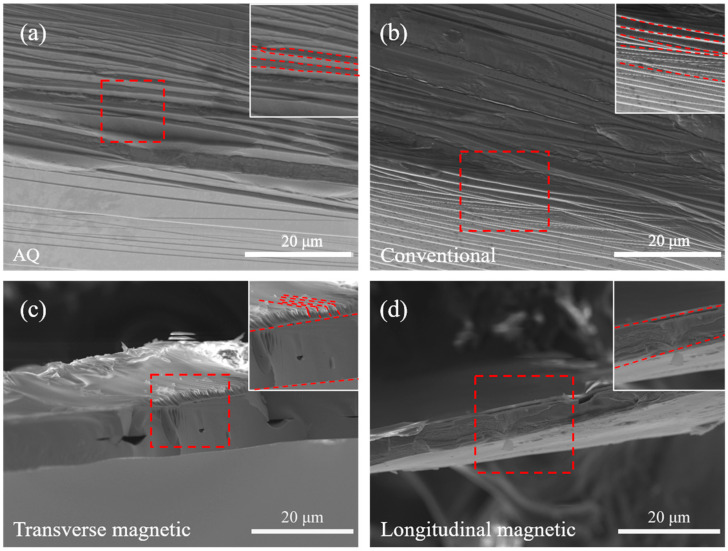
SEM images showing bent surface and fracture morphology of different samples.

At present, amorphous strips can achieve excellent soft magnetic properties and improve the microhardness of amorphous alloys by heat treatment, but at the same time, it also brings the defect of increased brittleness. As can be seen from the Figure 7 bending section SEM image, in addition to the transverse magnetic annealing fracture surface being very smooth, it shows obvious brittle fracture characteristics, while with the quenched state, conventional annealing and longitudinal magnetic annealing treatment of the fracture result in obvious fibrous toughness fracture characteristics. This indicates that the conventional annealing and longitudinal annealing of strips maintain better toughness, while seeming to obtain the best magnetic properties at the same time.

By studying the law of change of indenter load with indentation depth, we can understand the ability of the microstructure of the material to resist the deformation of external forces. The relationship between the load–combined displacement is obtained by fitting the unloading curve [24]:


(1) $$P=\alpha {\left(h-h_{f}\right)}^{m}$$

In the above equation, *P* is the load applied to the sample by the indenter; *h* is the indentation depth of the indenter; and *α* and *m* are the fitting parameters. The contact stiffness *S* is derived from h of the following equation:


(2) $$S=\left(\frac{dp}{dh}\right)h=h_{max}=\alpha m{\left(h_{max}-h_{f}\right)}^{m-1}$$

The contact area *C* of the Berkovich indenter can be obtained by the empirical formula:


(3) $$A=24.56h_{c}^{2}+\sum_{i=1}^{S}C_{i}h_{c}^{{\frac{1}{2}}^{i-1}}$$

In this equation, $C_{i}$ represents a coefficient to be determined, related to the indenter. The contact depth, $h_{c}$, can be obtained by using the following equation:


(4) $$h_{c}=h_{max}-\theta \frac{P_{max}}{S}$$

In the above equation, $P_{max}$ is the maximum load pressed in by the indenter; $h_{max}$ is the maximum displacement; *θ* is a constant associated with the indenter; and the *θ* of the Berkovich indenter is about 0.72. The contact area *A*, from which the hardness and elastic modulus of the material can be calculated, is then:


(5) $$H=\frac{P_{max}}{A_{max}\left(h_{c}\right)}$$

(6) $$E_{r}=\frac{\sqrt{\pi }}{2\beta }\frac{S}{\sqrt{A_{max}\left(h_{c}\right)}}$$

In the above equation, $E_{r}$ is the approximate modulus and is the shape constant of the indenter. The shape parameter of the Berkovich indenter used in this paper is 1.034.

**Figure 8:**
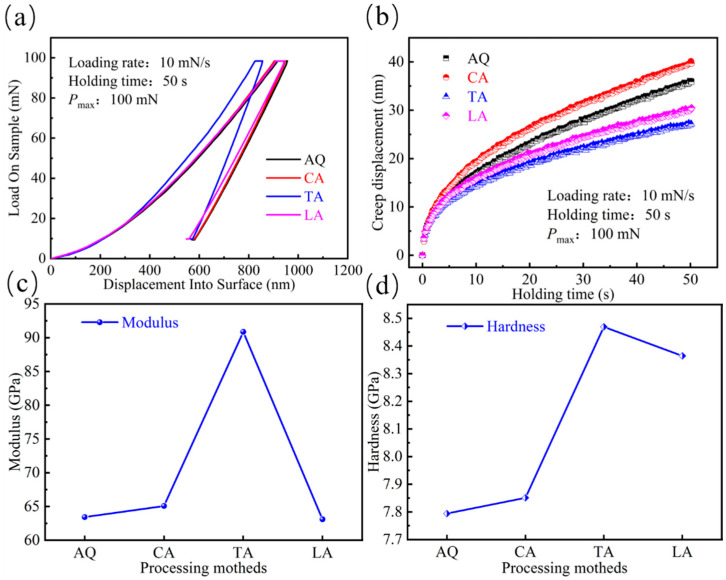
Mechanical properties of different samples: (**a**) Typical load–displacement curves of the as-quenched strip and the strip after different annealing treatments; (**b**) Curves of creep displacement versus holding time; (**c**) Comparison chart of Young’s modulus; (**d**) Comparison chart of hardness.

The load–displacement curves, shown in Figure 8a, show that the indenter can penetrate the magnetically annealed strip much shallower than the other three. It is evident from the figure that the indenter can penetrate the strip that underwent transverse magnetic annealing to a lesser extent than the other three. Furthermore, it has been documented in the literature [25] that the slope of the load–displacement curves is indicative of the material’s stiffness. This suggests that the transverse magnetically annealed strips exhibit higher levels of hardness and stiffness, accompanied by a degree of relaxation hardening.

When the indentation depth exceeds 400 nm, the load-bearing capacity of the transverse magnetic field annealed (TA) sample becomes significantly higher than the other three groups, and it exhibits a greater residual displacement recovery rate during the unloading phase. This phenomenon indicates that the TA sample possesses enhanced resistance to deformation during the plastic deformation stage, manifesting as a certain degree of relaxation hardening. This is mainly due to TA treatment-induced short-range ordering of Fe and Ni atoms, leading to a decrease in the free volume. Notably, the unique behavior of the TA sample during unloading suggests that mechanical loading may trigger localized microstructural transitions. Combined with the literature [26], the clusters induced by transverse magnetic field annealing might form brittle fracture paths under high-stress conditions. Meanwhile, the higher recovery rate of the LA sample during unloading could be attributed to magnetic field-induced atomic ordering. In longitudinal magnetic field annealing, atoms align along the magnetization direction, forming locally anisotropic structures. These structures release stored elastic energy through magnetoelastic coupling effects during unloading, promoting atomic recoil and reducing residual displacement. In contrast, the amorphous matrices of AQ and CA samples lack ordered structures, making plastic deformation recovery difficult after unloading.

In order to investigate the relaxation hardening in the load displacement, the treatment analysis of the load-holding phase in the nanoindentation test was carried out to investigate the changes in the internal structure of the strip after the annealing treatment. From Figure 8b, the following pattern of maximum creep displacement is observed: normal annealed (CA) > quenched state (AQ) > longitudinal annealed (LA) > transverse annealed (TA).

The maximum creep strain of the TA sample treated by magnetic field annealing was reduced by 40% compared to the CA sample, confirming a significant improvement in creep resistance. The research findings indicate that the performance differences between the LA and TA samples originate from microstructural regulation mechanisms: In LA samples, A1 FeNi nanocrystals synergistically enhance creep resistance through diffusion-suppression mechanisms (delaying viscous flow in quasi-liquid zones). Conversely, the presence of extensive atomic clusters within TA samples induces localized stress concentration, promoting microcrack nucleation and offsetting the material’s high hardness advantage. Additionally, short-range diffusion may accelerate localized plastic deformation processes. Combined with the brittle fracture morphology shown in Figure 8, this confirms that atomic cluster structures enhance hardness but weaken creep resistance by restricting energy dissipation pathways.

As demonstrated in Figure 8c,d, the hardness and Young’s modulus demonstrate analogous trends, thereby corroborating the preceding outcomes of the load–displacement curves. The transverse magnetically annealed strip exhibits the highest values for both Young’s modulus and hardness, with respective measurements of 90.8 GPa and 8.5 GPa. The elevated values of Young’s modulus and hardness suggest the presence of an accumulation of atoms within the strip or the formation of grains that precipitate and grow, thereby impeding the diffusion and reorganisation of atoms within the liquid-like region. Similar to conventional annealing, the thermal energy during magnetic field annealing promotes atomic diffusion motion, driving structural relaxation in the material. This process reduces the excess free volume and increases the atomic packing density, thereby enhancing the material’s hardness and strength. However, it is typically accompanied by a decline in toughness, as the nucleation and propagation of shear bands require higher stress, leading to more localized deformation. Nevertheless, when a longitudinal magnetic field is applied during annealing, it induces the alignment of magnetic moments along the field direction, generating an effective uniaxial magnetic anisotropy in the matrix. This magnetically induced anisotropy partially counteracts the negative impact of relaxation on plasticity by controlling the propagation direction of shear bands—orienting them parallel to the band length. As a result, the material may even exhibit relatively improved toughness.

**Figure 9:**
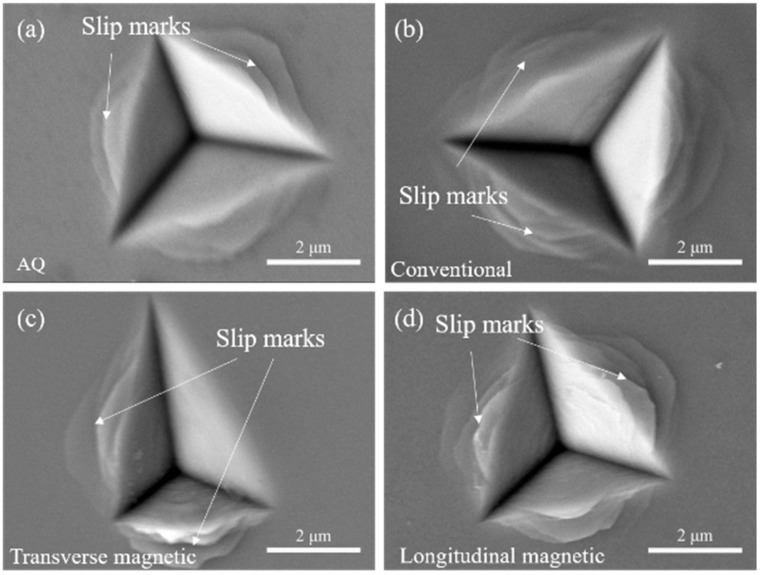
SEM images of nanoindentation obtained from different samples: (**a**) As-quenched (AQ); (**b**) Conventional annealing (CA); (**c**) Transverse magnetic annealing (TA); (**d**) Longitudinal magnetic annealing (LA).

In Figure 9a,b, the indentation contact regions of the as-quenched and conventionally annealed samples appear relatively smooth, with plastic deformation primarily accommodated by a large number of relatively uniformly distributed shear bands propagating outward from the indentation edges. In contrast, the indentation contact regions of the magnetically annealed samples exhibit higher surface roughness, particularly in the TA samples, where Figure 9c reveals more pronounced surface undulations and possible microscale features. This increased roughness is closely related to the microstructural anisotropy induced by magnetic annealing. Under transverse magnetic annealing conditions, the tendency of magnetization alignment toward ordered states guides the directional development of short-/medium-range ordered structures at the atomic scale during annealing, potentially forming submicron-scale topographic features on the surface associated with anisotropy. This rough surface leads to uneven contact between the indenter and the sample, resulting in localized stresses significantly higher than the apparent contact stress, which greatly promotes the nucleation of shear bands beneath the indenter. Simultaneously, the magnetically induced structural anisotropy restricts the free propagation of shear bands, and the combination of localized high stress facilitates the nucleation of dense shear bands, causing plastic deformation to concentrate more intensely directly beneath the indentation and potentially inducing localized microcracks. The combined effect manifests as shallower but more irregularly shaped indentations, along with an increased tendency toward brittle fracture.

Additionally, the number of shear band slip steps observed around the indentations in the longitudinally magnetically annealed and conventionally annealed samples is significantly higher than in the as-quenched and transversely magnetically annealed samples. This reflects that the LA and CA samples triggered more shear bands to accommodate deformation during the indentation plastic deformation process. For the LA samples, the magnetic field-induced easy magnetization axis being perpendicular to the strip length may provide a relatively low-energy barrier path for shear bands to propagate in the direction perpendicular to the strip length, allowing more shear bands to form, as shown in Figure 9d. The “stacking” degree of the shear band slip steps primarily reflects the total amount and localization extent of plastic deformation—numerous, widely distributed slip steps indicate relatively dispersed deformation, whereas fewer, confined slip steps suggest highly localized deformation.

In contrast to prior investigations [21], our investigations show that longitudinal magnetic field annealing preferentially induces A1 FeNi nanocrystals in FeNi amorphous alloys, which is attributed to atomic diffusion pathways regulated by the magnetic field direction. Comprehensive nanoindentation creep data showed a reduction in the maximum creep displacement of magnetic field-annealed specimens compared to quenched specimens, suggesting that the electrolytic microstructure suppresses plastic deformation while maintaining toughness. This finding provides a new strategy for the development of non-ferromagnetic alloys with synergistically enhanced soft magnetic properties and high toughness, bridging the gap in optimizing mechanical properties through magnetic annealing systems.

## 4. Conclusions

In this paper, the FeNiCBCo alloy, prepared by the single-roll fast quenching method, was subjected to magnetic field annealing in two different directions. Following this, a range of analytical techniques were employed to assess the alloy’s properties, including X-ray diffraction analysis, TEM analysis, soft magnetic property testing, and nanoindentation mechanical property testing. The results of this study were then compared with those obtained from the quenched state and conventional annealed samples. The following conclusions were obtained:

1. The strips all retained obvious amorphous features after magnetic field annealing, and α-Fe and A1 FeNi nanograins with a face-centered cubic structure were precipitated inside the longitudinal magnetically annealed samples.
2. The *H*_c_ of the transverse magnetic annealed sample is lower than that of the quenched state due to the substantial induced anisotropy within. In the material, although higher than that of the sample treated by normal annealing, the *H*_c_ reached a minimum of 0.27 A/m after the longitudinal magnetic annealing treatment, indicating that longitudinal magnetic annealing is better able to reduce the *H*_c_ of an FeNiCBCo alloy.
3. Through nanoindentation and SEM analyses, the transverse magnetic treatment led to the accumulation of magnetic atom pairs, which increased the brittleness and hardness of the strips and deteriorated their properties, while the longitudinal magnetic treatment induced the precipitation of A1 FeNi nanograins with better toughness inside the samples, which improved the hardness and toughness of the materials.
4. The FeNiCBCo alloy has been shown to enhance soft magnetic properties through the process of high-temperature longitudinal magnetic annealing, while also maintaining adequate toughness.

## Data Availability

The original contributions presented in this study are included in the article. Further inquiries can be directed to the corresponding author(s).
